# Mechanical versus manual chest compressions for out-of-hospital cardiac arrest: a meta-analysis of randomized controlled trials

**DOI:** 10.1038/srep15635

**Published:** 2015-10-27

**Authors:** Lu Tang, Wan-Jie Gu, Fei Wang

**Affiliations:** 1 Department of Anesthesiology, General Hospital of Jinan Military Command, Jinan, China; 2 Department of Anesthesiology, Affiliated Drum Tower Hospital, Medical College of Nanjing University, Nanjing, China

## Abstract

Recent evidence regarding mechanical chest compressions in out-of-hospital cardiac arrest (OHCA) is conflicting. The objective of this study was to perform a meta-analysis of randomized controlled trials (RCTs) to compare the effect of mechanical versus manual chest compressions on resuscitation outcomes in OHCA. PubMed, Embase, the Cochrane Central Register of Controlled Trials, and the ClinicalTrials.gov registry were searched. In total, five RCTs with 12,510 participants were included. Compared with manual chest compressions, mechanical chest compressions did not significantly improve survival with good neurological outcome to hospital discharge (relative risks (RR) 0.80, 95% CI 0.61–1.04, *P* = 0.10; *I*^*2*^ = 65%), return of spontaneous circulation (RR 1.02, 95% CI 0.95–1.09, *P* = 0.59; *I*^*2*^ = 0%), or long-term (≥6 months) survival (RR 0.96, 95% CI 0.79–1.16, *P* = 0.65; *I*^*2*^ = 16%). In addition, mechanical chest compressions were associated with worse survival to hospital admission (RR 0.94, 95% CI 0.89–1.00, *P* = 0.04; *I*^*2*^ = 0%) and to hospital discharge (RR 0.88, 95% CI 0.78–0.99, *P* = 0.03; *I*^*2*^ = 0%). Based on the current evidence, widespread use of mechanical devices for chest compressions in OHCA cannot be recommended.

Out-of-hospital cardiac arrest (OHCA) claims hundreds of thousands of lives annually all over the world. Only 1% to 8% of victims of OHCA survive to hospital discharge^1^ and up to half of survivors have some level of brain damage that may or may not completely resolve^2^. In the early cardiopulmonary resuscitation (CPR), high-quality chest compressions (i.e., sufficient depth, rate, full recoil of the chest and avoidance of interruptions) are crucial to both cardiac and brain resuscitation^3^,^4^.

Mechanical compression devices have been developed to provide high-quality chest compressions without the interruptions and fatigue associated with manual chest compressions. At present, there are two main mechanical compression devices: a load-distributing band CPR (LDB-CPR) device that provides circumferential thoracic compressions and a piston-driven CPR (PD-CPR) device that provides sternal compressions. Preclinical and observational studies suggest that mechanical chest compressions may increase cerebral blood flow, coronary perfusion pressures and cardiac output to improve survival compared with manual chest compressions^5^,^6^.

So far, application of mechanical compression devices during CPR has been evaluated in several systematic reviews. In the settings of out-of-hospital and in-hospital cardiac arrest, a Cochrane systematic review suggested insufficient evidence on the effectiveness of mechanical versus manual chest compressions^7^. Recently, a meta-analysis of randomized and observational studies focusing on OHCA showed a significant improvement in return of spontaneous circulation (ROSC) rate with mechanical chest compressions but did not assess survival outcomes^8^. Since then, several large randomized controlled trials (RCTs) addressing this topic have been published with conflicting results^9^,^10^,^11^. Given the limitations of observational studies with respect to managing risk of bias, in order to provide the latest and solid evidence, we conducted a meta-analysis of RCTs comparing the effect of mechanical versus manual chest compressions on survival and neurological outcomes in participants with OHCA.

## Materials and Methods

Ethical approval and patient consent were not required since this was a meta-analysis of previously published studies. The present meta-analysis was performed according to the Preferred Reporting Items for Systematic Reviews and Meta-Analyses statement^12^.

### Literature search and selection criteria

Relevant articles were identified by searching PubMed, Embase, the Cochrane Central Register of Controlled Trials, and the ClinicalTrials.gov registry (up to March 18, 2015). Electronic searches were conducted using Exploded Medical Subject Headings and the appropriate corresponding keywords, including “Mechanical chest compression”, “load distributing band”, “piston driven compression”, “AutoPulse” and “Lucas”. No language restriction was imposed. Additionally, the reference lists of the original studies and previous review articles were hand-searched to identify other potentially eligible studies.

Two authors (Tang L and Gu WJ) independently assessed the eligibility of all studies identified in initial research. Studies meeting the following criteria were included: (1) population: adult participants with non-traumatic OHCA; (2) intervention: mechanical chest compressions; (3) comparison: manual chest compressions and (4) design: RCTs. Agreement regarding trial inclusion was assessed using the Cohen К statistic^13^.

### Data extraction and outcomes definition

Two authors (Tang L and Gu WJ) independently extracted the following data: first author, year of publication, study location, participant characteristics, mechanical compression device, resuscitation strategy, adverse events, and main outcomes using a standard form. The original authors were contacted if data needed clarification or were not presented in the publication. Any disagreement was resolved by discussion.

The primary outcome was survival with good neurological outcome to hospital discharge. Secondary outcomes included survival to hospital admission, survival to hospital discharge, ROSC and long-term (≥6 months) survival. Neurological outcome was assessed using the Cerebral Performance Category (CPC) score or the modified Rankin Scale (mRS) score. Good neurological outcome was defined as the CPC score ≤ 2^14^ or the mRS score ≤ 3^15^. Survival to hospital admission was defined as patient admission to the hospital without ongoing CPR or other artificial circulatory support. ROSC was defined as the presence of any palpable pulse, in the absence of chest compression, and detectable by manual palpation. When data on survival to hospital admission was not available, we extract data on 4-hour survival after successful ROSC. In case that survival to hospital discharge was not reported, data on survival to 30 days were extracted. With respect to long-term survival, data on survival to six months or more were extracted.

### Risk of bias and evidence grade assessment

Two authors (Tang L and Gu WJ) independently assessed risk of bias in included RCTs with the method recommended by the Cochrane Collaboration^16^. Disagreements were resolved by consensus. The quality of evidence for the outcome measures was evaluated using the Grading of Recommendations Assessment, Development and Evaluation (GRADE) approach^17^. A summary table was prepared using the GRADE profiler (GRADEpro, version 3.6).

### Statistical analysis

All analyses were on an intention-to-treat basis. Differences were presented as relative risks (RRs) with 95% confidence intervals (CIs) for dichotomous outcomes. The Mantel-Haenszel method with the random-effects model was used to calculate pooled RRs and 95% CIs. Heterogeneity was quantified using the *I*^*2*^ statistic and if the *I*^*2*^ value was greater than 50%, heterogeneity was considered to be substantial. A priori subgroup analysis was conducted according to mechanical compression devices (LDB-CPR and PD-CPR). Publication bias was assessed by visually inspecting a funnel plot. *P* < 0.05 was considered statistically significant. All statistical analyses were conducted using Review Manager software (version 5.3; Nordic Cochrane Centre, Cochrane Collaboration).

## Results

### Study identification

**Figure 1:**
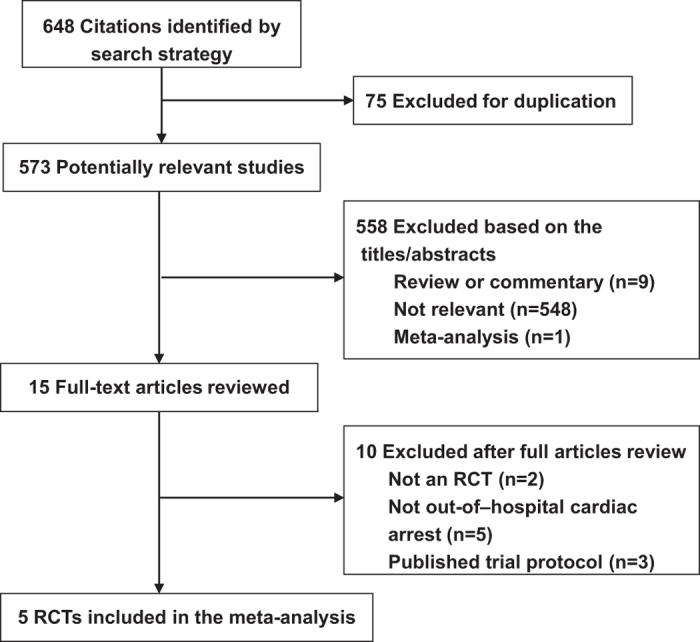
Selection of RCTs for the meta-analysis. RCT, randomized controlled trial.

The comprehensive search yielded 648 citations, of which 633 were eliminated for various reasons based on the title and abstract. The full texts of the remaining 15 publications were scrutinized for further evaluation. Finally, five RCTs^9^,^10^,^11^,^18^,^19^ with a total of 12,510 participants were included (Fig. 1). The Cohen statistic К for agreement on study inclusion was 0.93.

### Study characteristics

The main characteristics of the included RCTs are presented in Table 1. The trials were published between 2006 and 2015 and were all multicenter studies. The sample size ranged from 148 to 4471. Among the included trials, two^10^,^18^ compared LDB-CPR with manual CPR and the remaining three compared PD-CPR with manual CPR. Four^9^,^10^,^18^,^19^ included trials were sponsored by the manufacturer of the mechanical compression devices, and the remaining one trial^11^ was funded by National Institute for Health Research. Age, cardiac etiology, witnessing of cardiac arrest, bystander CPR and presenting rhythm were generally similar between the trial groups.

### Risk of bias assessment

**Figure 2:**
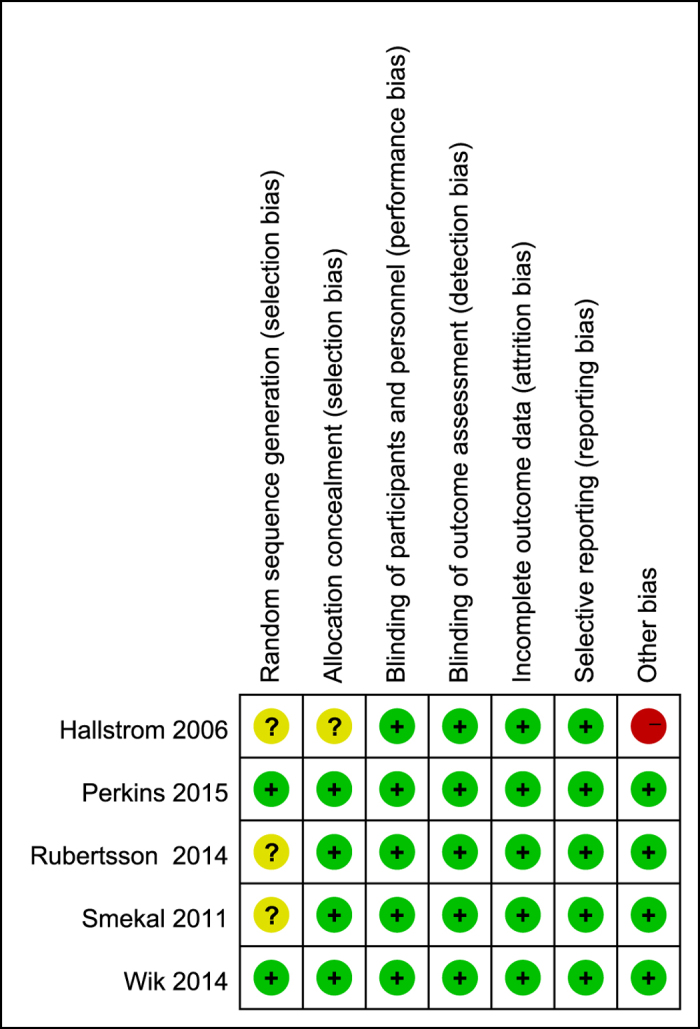
Risk of bias summary.

Randomized sequence was adequately generated in two trials^10^,^11^ and allocation sequence concealment was adequately reported in four trials^9^,^10^,^11^,^19^. Although blinding of participants and personnel and blinding of outcome assessors were difficult to implement due to the nature of the intervention, survival and neurological outcome assessment were very unlikely to have been influenced by knowledge of trial allocations. Thus, the two items (blinding of participants and personnel and blinding of outcome assessors) were judged to be low risk of bias for all the included trials. The numbers and reasons for withdrawal/dropout were detailed reported in all trials. One trial^18^ was defined as having other sources of bias because it was stopped early after the interim analysis. An overview of the risk of bias is summarized in Fig. 2.

### Primary outcome

**Figure 3:**
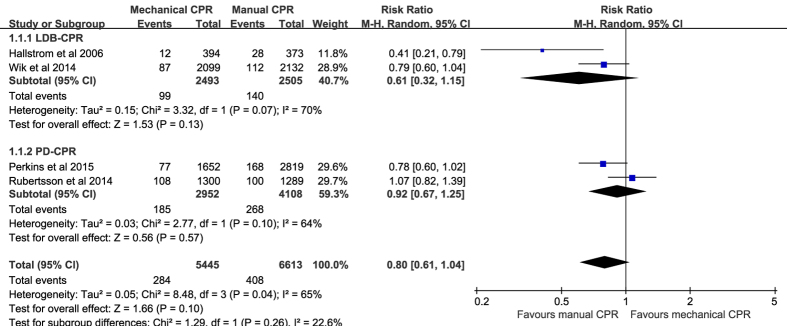
Forest plot of the effect of mechanical versus manual chest compressions on survival with good neurological outcome to hospital discharge.

Data on the primary outcome was available in four of the included RCTs (n = 12,058). Mechanical chest compressions did not significantly improve survival with good neurological outcome to hospital discharge (RR 0.80, 95% CI 0.61–1.04, *P* = 0.10; *I*^*2*^ = 65%; Fig. 3) compared with manual chest compressions. Subgroup analyses stratified by mechanical compression devices also suggested no significant difference in the primary outcome both in the LDB-CPR subgroup and in the PD-CPR subgroup.

### Secondary outcomes

**Figure 4:**
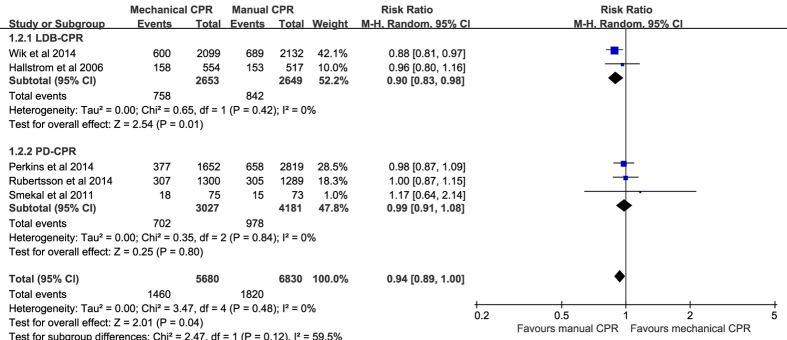
Forest plot of the effect of mechanical versus manual chest compressions on survival to hospital admission.

**Figure 5:**
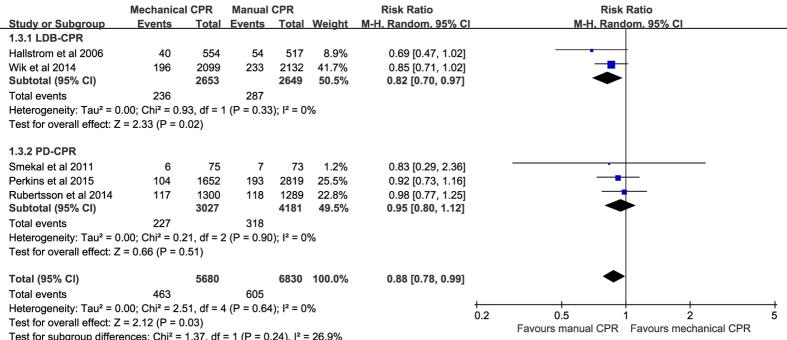
Forest plot of the effect of mechanical versus manual chest compressions on survival to hospital discharge.

Compared with manual chest compressions, mechanical chest compressions were associated with worse survival to hospital admission (RR 0.94, 95% CI 0.89–1.00, *P* = 0.04, *I*^*2*^ = 0%; Fig. 4), and to hospital discharge (RR 0.88, 95% CI 0.78–0.99, *P* = 0.03; *I*^*2*^ = 0%; Fig. 5). Subgroup analyses suggested worse survival to hospital admission and to hospital discharge were indicated in the LDB-CPR subgroup but not in the PD-CPR subgroup.

**Figure 6:**
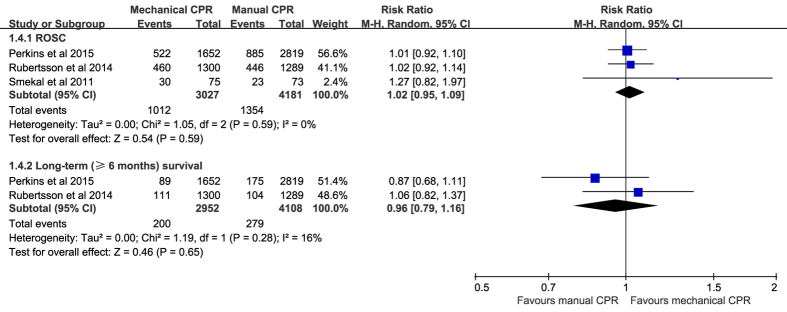
Forest plot of the effect of mechanical versus manual chest compressions on ROSC and long-term (≥6 months) survival. ROSC, return of spontaneous circulation.

No significant differences were observed in ROSC (RR 1.02, 95% CI 0.95–1.09; *P* = 0.59; *I*^*2*^ = 0%; Fig. 6) or long-term (≥6 months) survival (RR 0.96, 95% CI 0.79–1.16; *P* = 0.65; *I*^*2*^ = 16%; Fig. 6) between the mechanical CPR group and the manual CPR group.

### Quality of evidence and publication bias

The GRADE evidence profiles for the primary and secondary outcomes were shown in Table 2. The quality of evidence was low for survival with good neurological outcome to hospital discharge; moderate for survival to hospital admission, survival to hospital discharge, ROSC and long-term (≥6 months) survival.

For publication bias, the funnel plot was not conducted due to the small number of RCTs included.

## Discussion

The accumulated evidence from meta-analysis of RCTs suggested that mechanical chest compressions did not improve survival with good neurological outcome to hospital discharge, ROSC or long-term (≥6 months) survival, and were associated with worse survival to hospital admission and to hospital discharge in OHCA compared with manual chest compressions.

Differences between the current meta-analysis and a previous one by Westfall *et al.*^8^ should be noted. First, Westfall *et al.*^8^ conducted a meta-analysis of English-language randomized and non-randomized (phased, historical, and case-control) studies. Thereinto, only 2 small studies were RCTs^18^,^19^ and most of the included studies were non-randomized, thereby producing a potential for selection bias. Our meta-analysis included only RCTs and three large RCTs^9^,^10^,^11^ published since 2014 were included, which substantially enlarged the sample size (12,510 vs. 6,538 participants). Second, considering that survival data are deemed to be one of the highest quality and/or most clinically relevant endpoints for a given trial, we chose survival with good neurological outcome to hospital discharge, a more patient-centered outcome measure, as the primary outcome. Additionally, survival to hospital admission, survival to hospital discharge, ROSC and long-term (≥6 months) survival were also reported in the present meta-analysis. However, in Westfall *et al.*’s meta-analysis^8^, the primary outcome was ROSC and survival data were not reported. Third, the methods used for risk of bias assessment and GRADE profile evidence were described in our meta-analysis not in Westfall *et al.*’s^8^. Last, in Westfall *et al.*’s meta-analysis^8^, all authors have financial relationships with a manufacturer of mechanical compression devices, while in our meta-analysis, none of the authors have financial relationships with the manufacturers.

In the present meta-analysis, the findings of worse survival to hospital admission and to hospital discharge in participants receiving mechanical chest compressions were unexpected. One possible explanation is that interruptions in CPR during device deployment could cause reduced cardiac and cerebral perfusion. Furthermore, device deployment before the first defibrillation is likely to have led to a delay in the time to first defibrillation, which might in itself reduce survival. In one included RCT^18^, the time to first defibrillation was delivered 2.1 minutes later in the LDB-CPR group than in the manual CPR group. Similarly, in one included RCT evaluating the PD-CPR device^9^, the mechanical compression group had an average 1.5-minute extra delay to first defibrillation attempt. Therefore, if mechanical chest compression devices are being used in OHCA, special attention should be paid to minimizing the delay to chest compressions and defibrillation related to deployment of the device.

Since interruptions in CPR and delays in device deployment are a major factor that can impact outcomes, intensive and repetitive training is particularly important when using the mechanical compression devices in OHCA. In Perkins *et al.*’s study^11^, only 60% participants in the PD-CPR group received the allocated intervention and about 15% cases of non-use were because of difficulties inherent with implementation of new equipment and the training and quality issues associated with this. In a recent study^20^, mechanical chest compressions were associated with a higher no-flow ratio than manual chest compressions in the first five minutes of resuscitation. Where organizations decide to adopt mechanical compression device, it seems essential that sufficient resources are made available to support initial and regular refresher training and ongoing quality assurance.

Clinical adverse events occurring in chest compressions were reported in three included RCTs^9^,^10^,^11^, such as rib fractures, pulmonary edema, pneumothorax and chest bruising. Specifically, the type of device and the mechanism of the device were thought to be of paramount relevance to injury patterns observed in OHCA. A non-randomized study reported that the PD-CPR device slid from its original position, mostly in an abdominal direction during transport^21^. Although pooled analyses were not conducted due to significant clinical heterogeneity, no significant difference in serious adverse events was reported in individual included RCT.

The quality of evidence in the present meta-analysis ranked from moderate to low across the different outcomes. The limiting factor that was the reason for a decrease in quality for all outcomes was the serious risk of bias. Only two of the included RCTs were judged to be at low risk of bias. Additionally, due to the unexplained heterogeneity (*I*^*2*^ = 65%) across the small number of included studies, the evidence quality for the survival with good neurological outcome to hospital discharge was downgraded by one more level and was therefore assessed as low quality.

There are several limitations in the present study. First, although comprehensive literature search was conducted, the number of included RCTs was small and the funnel plot for publication bias was not conducted. Second, although more and more services emphasize the importance of ongoing performance monitoring for out-of-hospital CPR quality, none of the included RCTs reported any measures of CPR quality and we cannot assess the influence of the quality of manual CPR on the treatment effect estimates. Future studies comparing mechanical with manual CPR should include the instrumentation to monitor CPR quality. Third, although subgroup analyses suggested that LDB-CPR not PD-CPR significantly decreased survival to hospital admission and to hospital discharge than manual CPR, caution should be taken when interpreting the results because they were based on the pooled analysis of only two RCTs.

## Conclusions

Our meta-analysis suggested that mechanical chest compressions were not associated with better outcomes including survival with good neurological outcome to hospital discharge, survival to hospital admission, survival to hospital discharge, ROSC and long-term (≥6 months) survival in OHCA compared with manual chest compressions. Based on the current evidence, widespread use of mechanical devices for chest compressions in OHCA cannot be recommended.

## Additional Information

**How to cite this article**: Tang, L. *et al.* Mechanical versus manual chest compressions for out-of-hospital cardiac arrest: a meta-analysis of randomized controlled trials. *Sci. Rep.* **5**, 15635; doi: 10.1038/srep15635 (2015).

## Supplementary Material

Supplementary Information

## Figures and Tables

**Table 1:** Characteristics of included randomized controlled trials.

Study/year	Region	Comparison	No. of patients	Mean age (y)	Cardiac etiology (%)	Witnessed cardiac arrest (%)	Bystander CPR before EMS arrival (%)	VF/VT as initial rhythm (%)	Adverse events
Perkins *et al.*/2015^11^	4 UK Ambulance Services	PD-CPR vs M-CPR	4471	Mechanical: 71.0 ± 16.3 Manual: 71.6 ± 16.1	Mechanical: 86 Manual: 87	Mechanical: 61 Manual: 62	Mechanical: 43 Manual: 44	Mechanical: 23 Manual: 22	No serious adverse events were reported
Rubertsson *et al.*/2014^9^	4 Swedish, 1 British and 1 Dutch ambulance services	PD-CPR vs M-CPR	2589	Mechanical: 69.0 (16–100)* Manual: 69.1 (15–99)*	Mechanical: 65 Manual: 63	Mechanical: 66 Manual: 65	Mechanical: 57 Manual: 55	Mechanical: 29 Manual: 30	7 serious adverse events in the mechanical CPR group and 3 in the manual CPR group
Smekal *et al.*/2011^19^	2 Swedish cities	PD-CPR vs M-CPR	148	Mechanical: 69 ± 16 Manual: 71 ± 16	Not reported	Mechanical: 68 Manual: 74	Mechanical: 34 Manual: 31	Mechanical: 27 Manual: 27	Not reported
Wik *et al.*/2014^10^	3 US and 2 European sites	LDB-CPR vs M-CPR	4231	Mechanical: 65.7 ± 16.4 Manual: 65.6 ± 16.0	Mechanical: 100 Manual: 100	Mechanical: 37 Manual: 37	Mechanical: 47 Manual: 49	Mechanical: 21 Manual: 24	No significant difference between groups
Hallstrom *et al.*/2006^18^	United States and Canada	LDB-CPR vs M-CPR	1071	Mechanical: 66.6 ± 15.6 Manual:66.2 ± 15.2	Mechanical: 85 Manual: 86	Mechanical: 44 Manual: 49	Mechanical: 32 Manual: 35	Mechanical: 31 Manual: 32	Not reported.

Data are presented as mean ± SD unless indicated otherwise.

UK, united kingdom; US, united states; CPR, cardiopulmonary resuscitation; PD-CPR, piston-driven CPR; LDB-CPR, load-distributing band CPR; M-CPR, manual CPR; EMS, emergency medical systems; VF, ventricular fibrillation; VT, ventricular tachycardia.

^*^Mean (range).

**Table 2:** GRADE evidence profile.

Quality assessment																	No of patients				Effect					Quality	Importance
No of studies				Design			Risk of bias				Inconsistency	Indirectness	Imprecision			Other considerations	Mechanical chest compressions		Manual chest compressions		Relative (95% CI)		Absolute				
Survival with good neurological outcome to hospital discharge																											
4			randomized trials			serious^1^				serious^2^		no serious indirectness		no serious imprecision	none			284/5445 (5.2%)		408/6613 (6.2%)		RR 0.8 (0.61 to 1.04)		12 fewer per 1000 (from 24 fewer to 2 more)		LOW	CRITICAL
																				6.7%				13 fewer per 1000 (from 26 fewer to 3 more)			
Survival to hospital admission																											
5		randomized trials				serious^1^			no serious inconsistency			no serious indirectness		no serious imprecision	none			1460/5680 (25.7%)		1820/6830 (26.6%)		RR 0.94 (0.89 to 1)		16 fewer per 1000 (from 29 fewer to 0 more)	MODERATE		IMPORTANT
																				23.7%				14 fewer per 1000 (from 26 fewer to 0 more)			
Survival to hospital discharge																											
5	randomized trials				serious^1^			no serious inconsistency				no serious indirectness		no serious imprecision	none			463/5680 (8.2%)		605/6830 (8.9%)		RR 0.88 (0.78 to 0.99)		11 fewer per 1000 (from 1 fewer to 19 fewer)	MODERATE		IMPORTANT
																				9.6%				12 fewer per 1000 (from 1 fewer to 21 fewer)			
Return of spontaneous circulation																											
3	randomized trials				serious^3^			no serious inconsistency				no serious indirectness		no serious imprecision	none			1012/3027 (33.4%)		1354/4181 (32.4%)		RR 1.02 (0.95 to 1.09)		6 more per 1000 (from 16 fewer to 29 more)	MODERATE		IMPORTANT
																				31.5%				6 more per 1000 (from 16 fewer to 28 more)			
Long-term (≥6 months) survival																											
2	randomized trials				serious^3^			no serious inconsistency				no serious indirectness		no serious imprecision	none			200/2952 (6.8%)		279/4108 (6.8%)		RR 0.96 (0.79 to 1.16)		3 fewer per 1000 (from 14 fewer to 11 more)	MODERATE		IMPORTANT
																				7.1%				3 fewer per 1000 (from 15 fewer to 11 more)			

^1^Only two trials were judged to be at low risk of bias.

^2^Substantial heterogeneity (*I*^2^ = 65%) was found.

^3^Only one trial was judged to be at low risk of bias.
